# Different grain-filling rates explain grain-weight differences along the wheat ear

**DOI:** 10.1371/journal.pone.0209597

**Published:** 2018-12-31

**Authors:** Nadège Baillot, Christine Girousse, Vincent Allard, Agnès Piquet-Pissaloux, Jacques Le Gouis

**Affiliations:** 1 Vetagrosup, campus agronomique, Lempdes, France; 2 GDEC, INRA, UCA, Clermont-Ferrand, France; Institute of Genetics and Developmental Biology Chinese Academy of Sciences, CHINA

## Abstract

Thousand grain weight is one of the components determining wheat grain yield. It represents the average value of individual grain weights which depends on position within the ear and on positon within the spikelet. Our objective was to quantify the influences of individual floret anthesis date, of carpel weight at anthesis and of rate and duration of grain filling, on variation in individual final grain weight. Two bread wheat cultivars were grown in a greenhouse and their ears were sampled from anthesis through to harvest. Each ear was divided into three parts—basal, central and apical—where the two proximal grains were dissected from each of two spikelets. We analysed (i) the flowering time shift within the ear and within the spikelet; and (ii) the growth kinetics during grain filling in relation to position along the ear. For both cultivars, florets located in the central part of the ear were the first to reach anthesis followed by those in the apical part and then the basal part. Within a spikelet, the floret located nearest the rachis flowered first followed by the more distal ones. We found no significant systematic effect of flowering time-shift on final grain weight. Nevertheless, grains in the central part were heavier than the basal ones (9.75% smaller) and than the apical ones (18.25% smaller). These differences were explained mainly by differences in mean grain filling rates. Analysis of growth kinetics enabled an improved explanation of the variability of individual grain weight along the ear.

## Introduction

Grain yield of bread wheat (*Triticum aestivum L*.) can be decomposed into two main components: (i) the number of grains per square meter, which is determined before anthesis and (ii) the thousand grain weight (TGW), which is determined during grain filling. Although grain number per square meter has been identified as the main driver of yield variability [1, 2], there is an urgent need to better understand the sources of variation in TGW because climate change may well lead to increases in abiotic stress during grain filling (*e*.*g*. [3, 4]).

The TGW is generally calculated at the scale of a plot and represents the mean of many individual grain weights. However, wide variability exists in individual grain weight, and this variability has implications for commercial value [5]. It has been proposed that part of the genetic improvement of TGW may rely on a putative genetic variability associated with individual grain-weight distribution within the ear [6]. To pursue a strategy of genetic improvement of TGW based on individual grain-weight variation, requires a better understanding of the ecophysiological mechanisms associated with this variation.

The final grain weight is determined during grain filling, from anthesis to maturity. Grain filling is considered to occur during three sequential phases of grain development: cell division, grain dry matter accumulation and grain maturation and desiccation [7]. While the kinetics of grain filling has been described for a number of species, there is still debate as to whether the main driver for final grain weight difference, is the duration of grain filling or the rate of grain filling. Some authors have observed strong correlations between final grain weight and the duration of grain development—in corn [8, 9] and in durum wheat [10]. Conversely, for pearl millet [11], rice [12] and bread wheat [13, 14, 15, 16], differences in final grain weight have been attributed to differences in grain filling rate.

A weakness of some of these studies is they have focused on specific grains, generally located in the central part of the ear. They have not taken into account developmental variability within the ear and the possible effects of an associated grain-weight distribution. As already noted, significant variability exists in individual grain weight within the ear. This variability originates from two distinct gradients. First, it is generally true that spikelets in the central part of the ear tend to bear heavier grains than those in the basal and apical parts [17, 18, 19, 20]. Second, differences have also been observed when comparing proximal and distal grains in a spikelet [6, 15, 21]. In the spikelet, the grain weight gradient is also a matter of uncertainty. Some authors report that the second grain in a spikelet is heavier than the proximal grain and also than the more distal ones [13, 16, 17, 19, 20, 21, 22]. Conversely, Herzog and Stamp [20] found the proximal grain to be generally heavier than the one in the second position within a spikelet, while Feng et al. [6] reported no significant weight differences between these positions. However, most of the above studies were largely descriptive and did not propose candidate processes that might explain the observed differences in grain weight.

Reports that focus on the effects of grain spatial position within the ear on grain growth and employing kinetic growth analysis are scarce and they often lead to conflicting hypotheses. Miralles and Slafer [15] found that differences in final grain dry matter depended mainly on the rate of grain development and this varied with position in the ear. Bremner [18] and Herzog and Stamp [20] concluded that both the rate and the duration of grain development were influenced by grain position. Nevertheless, the methods used in these studies do not take into account processes that may be important. In particular, the flowering time of each floret and the carpel weight at anthesis were not taken into account as putative co-variables. For example, individual grain flowering time is subject to the same two gradients as described above. Thus Rawson and Evans [17] show that florets in the centre of the ear reach anthesis first, followed by the more distal florets, one day later, and then the more proximal florets, three days later. Moreover, within a spikelet, as many as four days can elapse between the anthesis of the first and last florets [17]. Moreover, carpel size also affects final grain weight, with carpel size within spikelets in the central part of the ear being positively correlated with final grain weight [13, 16, 22]. A recent study also showed that the larger the carpel, the faster the rates of grain growth and grain filling, these resulting in heavier grains [16]. However, these studies did not correlate spikelet position along the ear with carpel size. Carpel size differences may well be a cause of the grain weight differences found in different parts of the ear.

Thus, although the literature offers good information on the spatial variability of individual grain weight within the ear and on the growth dynamics of specific grains, it lack more detailed analyses of grain growth dynamics in different regions of the ear and of the spikelet. Only a few studies attempt this [15, 18, 20] but the methods did not allow robust statistical comparison to be made of the kinetic parameters of grains in the different ear parts.

Our objective was to identify the factors determining individual grain weight variation both along the ear and within the spikelet by measuring four major traits: the timing of floret anthesis, the carpel weight at anthesis, and the rate and duration of grain development. To do this we characterised the growth dynamics of grains located in different parts of the ear using two cultivars of greenhouse-grown bread wheat.

## Materials and methods

### Experimental design

We selected two winter wheat cultivars registered in France known to differ in grain yield and its components from a field experiment conducted in 2014 in Clermont-Ferrand, France (S1 Table). The first, Apache, is characterised by having higher values for grain yield, ear number per plant, grain number per ear and spikelet number per ear than the second, Renan, which is characterised by having higher values for TGW.

The experiment was conducted in a greenhouse between January and July 2017 at the INRA, Clermont-Ferrand (45°78' N, 3°08' E, 401 m a.s.l). Two weeks after germination, plants were vernalised for eight weeks at 5°C before transplanting. Three plants of the same cultivar were transplanted into 4 L plastic pots (18.8 cm diameter × 21.8 cm height) filled with a compost enriched with 2.5 kg m^-3^ of fertiliser 9-12-16 (N-P-K), and iron (Fe).

Plants were grown at regulated day/night air temperatures of 18/15°C and under natural daylight supplemented to a photoperiod of 16 h of daylight using 400 W sodium vapour lamps. Water and nutrient solution were provided to excess twice a day. From transplanting to maturity, air temperature, relative humidity and photosynthetically active radiation (PAR) were recorded every fifteen minutes using eight CR 1000 dataloggers (Campbell Scientific, Logan, UT, USA) placed uniformly within the greenhouse to capture any putative environmental gradients. Environmental data during the experiment are presented in S1 Fig.

The trial was arranged in a block design, each of the four blocks consisting of 52 pots (26 pots per cultivar).

### Plant sampling and measurements

#### Flowering time along the ear

Ten plants per cultivar with the same number of spikelets on their main stems (n = 22 for Apache and n = 20 for Renan) taken from the four blocks were retained to study the flowering times of the various florets according to the positions of the spikelets within the ears and of the florets within the spikelets. The flowering time of each floret was recorded as when the three anthers were extruded. The position of each spikelet within the ear was recorded by numbering sequentially from base to apex. Within a spikelet, the first floret was that nearest the rachis and the last (3^rd^ or 4^th^ depending on the cultivar) was the most distant.

To allow comparison between ears, the flowering time of each floret was standardised by expressing it as a thermal time difference (°Cdays; base temperature 0°C) relative to the flowering time of the earliest floret of the ear. These values were averaged over the ten replicates (ten ears per cultivar).

#### Grain dry matter accumulation

During booting, the main tiller of each plant was identified and tagged. At ear emergence, the spikelet number of each tagged ear was recorded. To minimise variability linked with ear size variation, only plants that displayed the median number of spikelets on the main tiller for each cultivar were selected and used for the rest of the study. For Renan, plants with 20 spikelets per ear were selected, while for Apache (to reach a sufficient sample size) plants with 21 or 22 spikelets per ear were selected.

**Fig 1:**
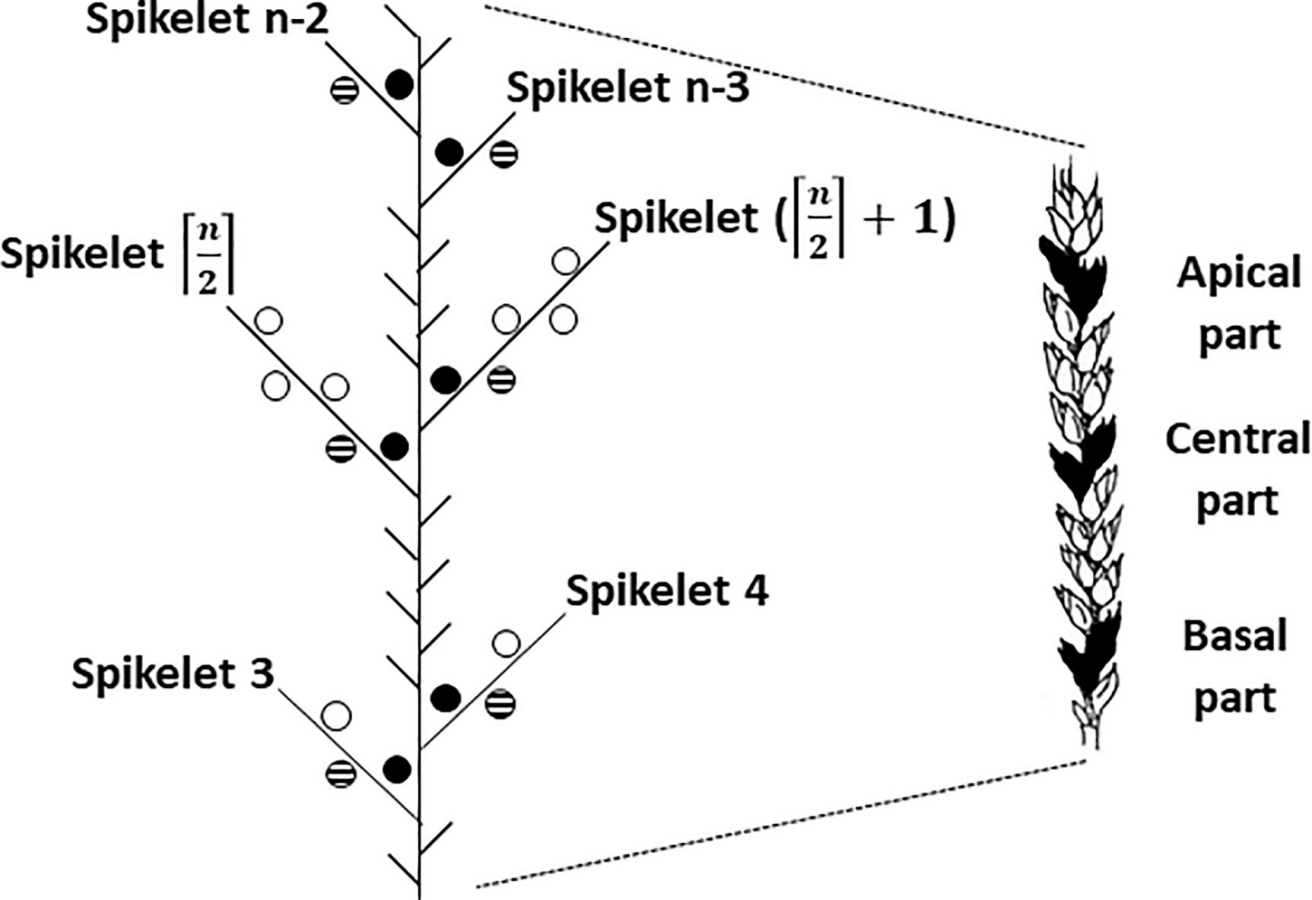
Schematic of a wheat ear at maturity indicating the positions of the spikelets and grains studied. Black circles: grain in first position; striped circles: grain in second position; open circles: other grains. n indicates the total number of spikelets.

The dynamics of grain filling was studied for grains that belonged to spikelets in specific parts of the ear. Ears were considered to contain three parts—basal, central and apical. Each part was represented by two spikelets. For a given number of spikelets per ear (n), and numbering spikelet positions from ear base to apex, ear parts were defined as (Fig 1):

basal = 3^rd^ and 4^th^ spikelets;
central = ${\left\lceil \frac{n}{2}\right\rceil }^{\mathrm{th}}$ and ${\left(\lceil \frac{n}{2}\rceil +1\right)}^{\mathrm{th}}$ spikelets;
apical = (n-3)^th^ and (n-2)^th^ spikelets.

Grain filling dynamics were studied by carrying out a number of samplings from anthesis to maturity. To obtain a clear view of the initial state of the grains, and because flowering time varied between ear parts, the first sampling (at 0°Cdays) was specific to each part. Three separate samples were taken when florets in the central, apical and basal parts reached anthesis, respectively. Each sample was made up from eight tagged ears, by collecting the two proximal grains, of the two spikelets, of each ear part.

After anthesis, subsequent samplings were carried out about every four days until maturity. Grains were collected at 17 different developmental stages for Apache and at 13 for Renan. These sampling were again made on eight tagged ears per cultivar and of grains collected as above. After sampling, each grain was oven dried to constant weight at 60°C for two days and weighed with a precision balance with 0.001 mg precision (Mettler-ToledoAX205 DR, Switzerland).

#### 2.2.3 Statistical analysis

For each grain position in the ear, the different grain-filling parameters were estimated by fitting a three-parameter logistic model, which has been demonstrated as an adequate model to describe the grain development [23]:

$$Y=\frac{Y_{m}}{1+(\frac{Y_{m}-Y_{0}}{Y_{m}}){exp}^{-\mu t}}$$ (Eq 1)

Where *Y* is the individual grain dry weight (mg), *Y*_*m*_ is the final grain weight (the upper asymptote), *Y*_*0*_ is the grain weight at flowering (the lower asymptote) and *𝜇* is a time constant parameter (°Cdays^-1^). Time *t* was expressed as the thermal time after flowering (°Cdays).

The duration of grain filling, *D* (°Cdays) was calculated by solving the Eq 1 when *Y* reached 95% of the upper asymptote (*Y*_*m*_). Therefore, *D* was calculated as:

$$D=ln\left(19(\frac{Y_{m}}{Y_{0}}-1)\right)$$ (Eq 2)

The mean grain filling rate, *R*_*mean*_ (mg.°Cdays^-1^) was then calculated by dividing the estimated final grain dry weight from Eq 1 by the grain-filling duration calculated from Eq 2:

$$R_{mean}=\frac{Y_{m}}{D}$$ (Eq 3)

The maximal grain filling rate, *R*_*max*_ (mg.°Cdays^-1^) was equal to the first derivative of Eq 1 at the time which the second derivative was equal to 0. It was calculated by solving the following equation as described by Robert et al. [23]:

$$R_{max}=\frac{\left(Y_{m}\times \mu \right)}{4}$$ (Eq 4)

For each cultivar, analyses of variance (ANOVA) were carried out separately to study the effects of spikelet position (basal, central and apical) on the different grain filling parameters. The ANOVAs were carried out using the mixed-effects model:

$$Y_{ij}=\mu +{EP}_{i}+b_{j}+\epsilon_{ij}$$ (Eq 5)

Where *Y*_*ij*_ is the trait value for ear part *i* in block *j*, *μ* is the general mean, *EP*_*i*_ is a fixed factor representing the different parts of the ear, *b*_*j*_ a random factor representing the block and *ϵ*_*ij*_ is the residual error term. After each ANOVA, multiple comparison tests (Newman-Keuls) were carried out. To explore correlations between grain filling parameters and final grain weight, Pearson correlation coefficients were calculated.

Similarly, the effects on flowering time of grain positions within-ear and within-spikelet were explored by ANOVA using the mixed-effects model:

$$Y_{ijk}=\mu +SP_{i}+GP_{j}+SP_{i}\times {GP}_{j}+b_{k}+\epsilon_{ijk}$$ (Eq 6)

Where *Y*_*ijk*_ is the trait value for grain *j* in spikelet *i* and block *k*, *μ* is the general mean, *SP*_*i*_ is the first fixed factor representing the spikelet position within the ear, *GP*_*j*_ is the second fixed factor representing the grain position within the spikelet, *b*_*k*_ is a random factor representing the block and *ϵ*_*ijk*_ is the residual error term.

All statistical analyses were carried out using the computing environment R v3.4.1 (R Core Team, 2017).

## Results

### Yield components of the two cultivars

The two cultivars, Apache and Renan, were chosen because a previous field experiment revealed they possessed contrasting yield components. The mean values of the yield components for the two cultivars under field conditions and in the present greenhouse study are shown in S1 Table. Under field conditions, compared with Renan, Apache had significantly more spikelets and grains per ear but a lower TGW. The cultivar differences were less marked under greenhouse conditions, with the exception that the number of ears per plant was increased, likely due to a higher plant density. Nevertheless, the trends were similar. Thus, we believe these two cultivars offer a useful example of contrasting grain-set strategies and this may help expand the usefulness of the results.

### Flowering pattern along the ear for the two cultivars

To obtain a synoptic understanding of the flowering pattern at the ear scale, flowering time was recorded individually for all florets of ten main ears of the two cultivars. Note that the number of fertile florets varied between positions and with genotype (S2 Fig). The maximum numbers of florets per spikelet were four for Apache and three for Renan. Moreover, the numbers of spikelets where a third floret was present was lower in Renan than in Apache. These two traits led to Renan bearing a smaller number of potential grains. As expected, the number of florets per spikelet decreased drastically from the central part of the ear to the basal and apical parts (S2 Fig).

**Fig 2:**
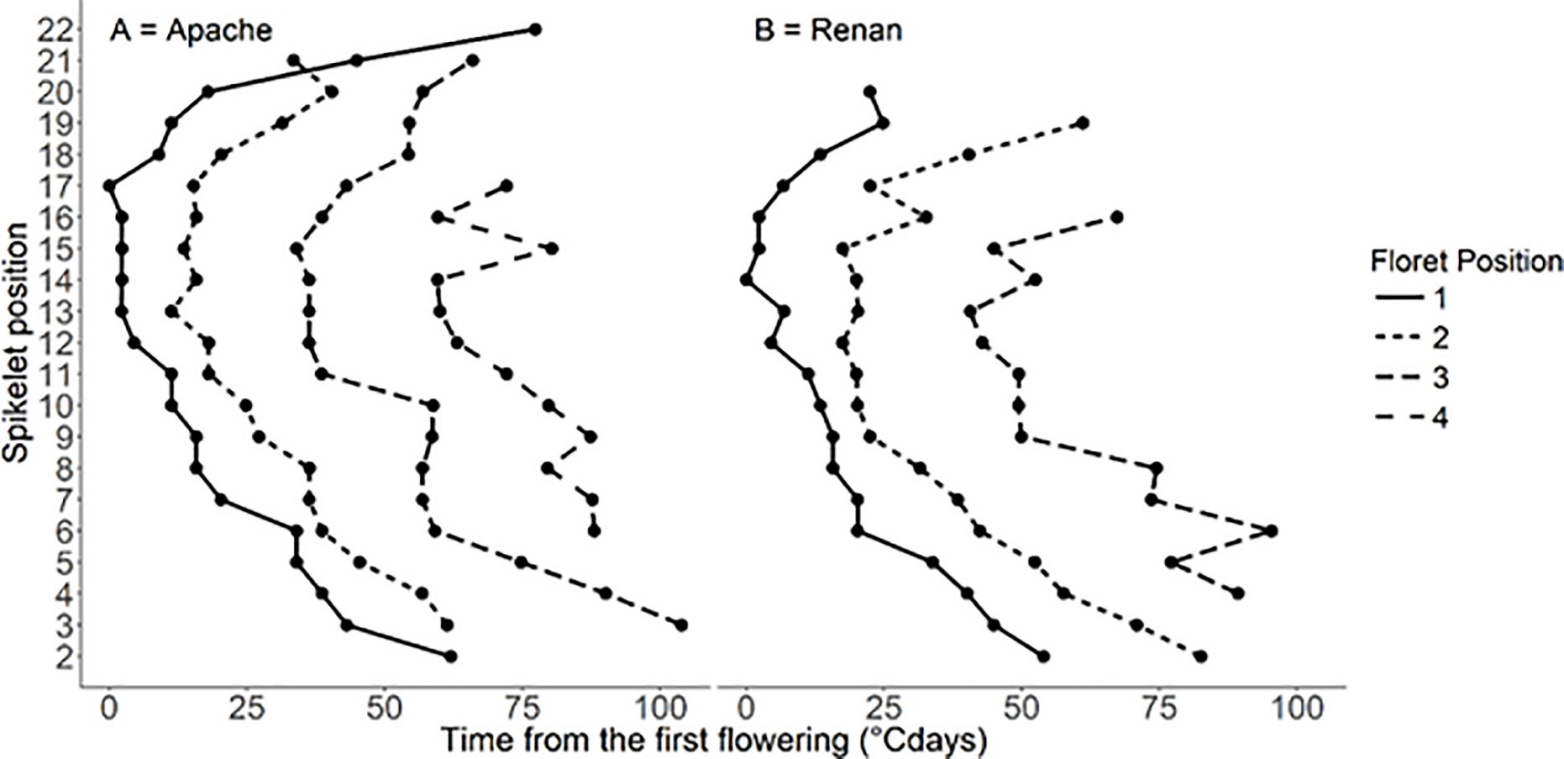
**Average flowering time shift in relation to spikelet position in the ear and to floret position within the spikelet for cultivars Apache (A) and Renan (B).** Position in the ear is numbered from base to tip and position within the spikelet is numbered from nearest to the rachis to the most distant. For each cultivar, ten ears were observed with 22 spikelets for Apache and 20 spikelets for Renan. Each dot represents the mean of observed values. °Cdays is thermal time (base 0°C).

Average profiles of flowering time for each floret position within the ear and within the spikelet are shown in Fig 2. The spatial flowering pattern followed two gradients.

First, in both cultivars, spikelet position had a strong effect on flowering time. Spikelets inserted in the upper central part of the ear (17^th^ for Apache and 14^th^ for Renan) were the first to reach anthesis. Spikelets inserted at the ear apex were first to reach anthesis (about 15°Cdays^-1^ later) while those at the base (from 2^nd^ to 6^th^ spikelet for Apache and 2^nd^ to 5^th^ spikelet for Renan) were the last to reach anthesis (about 40°Cdays^-1^ later).

Second, a further gradient was present at spikelet level. Independent of spikelet position, the proximal florets (nearest the rachis within a spikelet) were the first to reach anthesis while the distal florets showed progressively delayed flowering (Fig 2). On average for Renan, over all spikelet positions, the delay was 18°Cdays^-1^ for floret 2 and 48°Cdays^-1^ for floret 3. For Apache the delays were 14°Cdays^-1^ for floret 2, 40°Cdays^-1^ for floret 3 and 65°Cdays^-1^ for floret 4.

In Apache, anthesis occurred over a 3.5 day period, with an interval of 2.0 days between the anthesis of the first floret in each spikelet and that of the last. In Renan, the period was 3.0 days and the interval was 1.6 days.

As described above, the dynamics of grain growth as the accumulation of dry matter per grain was not recorded for all grains but only for the proximal two grains (1^st^ and 2^nd^) of spikelets inserted in the basal, distal and apical parts of the ear. The presence of grains in the 3^rd^ and 4^th^ positions in the spikelet (Apache) and in the 3^rd^ position (Renan) was not sufficiently common in the basal and apical parts of the ear for their growth to be analysed. For consistency with this sampling pattern, the flowering time dynamics was analysed following this same pattern. An ANOVA was carried out for each cultivar but only on the two proximal florets within the two spikelets (i.e. four florets) in the basal, central and apical parts of the ear. The results show flowering time is significantly related to spikelet position (p<0.01 for Apache and p<0.001 for Renan) and to floret position (p<0.05 for both cultivars) within the ear. No significant interaction was detected which indicates the two gradients are simply additive in their effects. In Apache, florets inserted in the central part reached anthesis 13°Cdays (about 0.6 days) earlier than those in the apical part (Table 1). Florets in the basal part were the last to reach anthesis 37.1°Cdays (or 1.6 days) later than those in the central part. In Renan, florets in the central and apical parts reached anthesis 33.7°Cdays (about 1.5 days) earlier than those in the basal part (Table 1). Within a spikelet, the 1^st^ floret reached anthesis 16.6°Cdays (less than 1 day) earlier than the 2^nd^ floret in Apache and 16.5°Cdays earlier in Renan (Table 1).

**Table 1:** Anthesis time shift (means and standard deviations) of the three different ear parts and of the floret positions within the spikelet for each wheat cultivar.

Time shift (°Cdays)	Apache					Renan				
	Ear part			Floret Position		Ear part			Floret Position	
	Basal	Central	Apical	1^st^	2^nd^	Basal	Central	Apical	1^st^	2^nd^
											Mean	52.9^c^	15.8^a^	28.4^b^	24.1^a^	40.6^b^	51.1^b^	15.0^a^	19.7^a^	19.2^a^	35.7^b^
*SD*	*15*.*5*	*12*.*9*	*32*.*0*	*22*.*7*	*27*.*8*	*18*.*3*	*12*.*6*	*21*.*4*	*18*.*3*	*25*.*7*

^a,b,c^Means with the same letter within a row for each factor are not significantly different according to a Newman-Keuls post-hoc test following an ANOVA with ten replicates. The first floret to reach anthesis was set to 0 and the numbers of degree-days (°Cdays, base 0°C) were summed for the other florets on the same ear as they reached anthesis.

### Grain growth traits according to grain position along the ear

As explained above, we followed only the two proximal grains in the six spikelets. Independent of position in the spikelet, no significant differences in final grain weight were observed between the two proximal grains (S3A Fig). Therefore, the two proximal grains were pooled in subsequent analyses, that focus on the gradient along the ear.

For both cultivars, the three ear parts and the four experimental blocks, the parameters of grain filling were estimated from a three-parameter logistic. The synoptic model provided a good fit to the data with R^2^ ranging from 0.76 to 0.98. The graph of residuals (S4 Fig) showed some heteroscedasticity, with an increase in residual variance as grain growth proceeded. The logistic equation also somewhat overestimated the initial grain weight at anthesis (S5 Fig). Consequently, we decided to use the observed initial grain weight rather than the predicted one. Therefore, Eq 1 was used to estimate final grain weight and to calculate the average and maximum grain-filling rates as well as the durations of grain filling.

**Fig 3:**
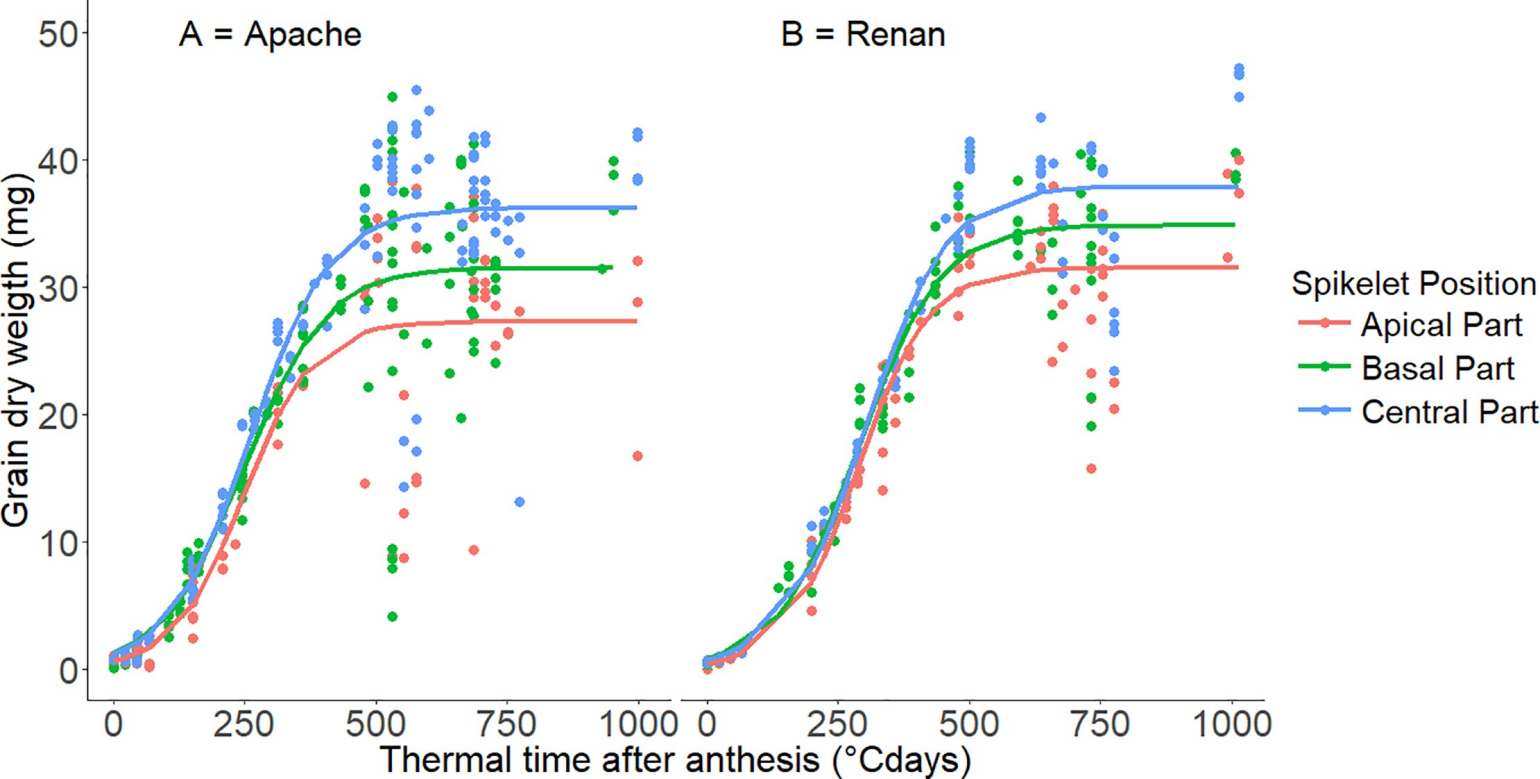
The relationship between grain dry weight and thermal time after anthesis. Data are taken from the 3^rd^ block for the three different ear parts (light-grey circles: apical part, dark-grey circles: basal part, black circles: central part) and for cultivars Apache (A) and Renan (B). °Cdays is thermal time (base 0°C).

Fig 3 illustrates the accumulation of dry matter per grain from anthesis to maturity for the third block, the three ear parts and for Apache and Renan. The fits obtained for the other blocks are presented in supplementary data (S5 Fig). For each cultivar, ANOVAs were carried out for all parameters to identify potential effects of position within the ear. For both cultivars, all parameters estimated were affected by ear position except the duration of grain filling (Table 2). Averaged over the three ear parts, the duration of grain filling was 498°Cdays for Apache and 567°Cdays for Renan. In both cultivars, final estimated grain weights were significantly larger in the central ears parts. Meanwhile, in Apache, the final estimated grain weights were 11.2% smaller in the basal part and 25.3% smaller in the apical part, and in Renan the final estimated grain weights were 8.3% smaller in the basal part and 11.2% smaller in the apical part. Similarly, higher values of mean grain-filling rate were observed in the central part for both cultivars. For Apache, the mean rate of grain growth was 13.0% slower in the basal part and 20.3% slower in the apical part and, for Renan, it was 9.5% slower in the basal part and 13.5% slower in the apical part. In both cultivars, the maximal grain filling rate was greater in the central part of the ear, than in the basal or apical parts (Table 2). Grain initial weight measurements highlighted different behaviours between the two cultivars. In Apache, *Y*_*0*_ was 40.3% higher in the central part of the ear than in the basal or apical parts, while in Renan it was 38.4% higher in central and basal parts than in the apical part (Table 2).

**Table 2:** Effects of ear parts on the parameters describing grain growth for cultivars Apache and Renan.

Grain filling parameters	Apache				Renan			
	F(test)	Basal	Central	Apical	F(test)	Basal	Central	Apical
Y_0_ observed (mg)	***	0.43^b^	0.74^a^	0.47^b^	**	0.97^a^	1.0^a^	0.79^b^
*SD*		*0*.*31*	*0*.*2*	*0*.*21*		*0*.*57*	*0*.*19*	*0*.*21*
Y_m_ estimated (mg)	***	30.9^b^	34.9^a^	26.1^c^	***	40.3^b^	43.5^a^	37.0^c^
*SD*		*2*.*3*	*2*.*5*	*3*.*4*		*6*.*1*	*5*.*2*	*5*.*5*
Duration (°Cdays)	ns	513^a^	504^a^	467^a^	ns	599^a^	583^a^	573^a^
*SD*		*63*	*45*	*31*		*86*	*48*	*76*
Mean rate (mg.°Cdays^-1^)	***	0.061^b^	0.07^a^	0.056^c^	***	0.067^b^	0.074^a^	0.065^c^
*SD*		*0*.*008*	*0*.*007*	*0*.*007*		*0*.*008*	*0*.*003*	*0*.*001*
Maximal rate (mg.°Cdays^-1^)	**	0.094^b^	0.111^a^	0.095^b^	**	0.106^b^	0.120^a^	0.105^b^
*SD*	* *	*0*.*033*	*0*.*028*	*0*.*023*	* *	*0*.*01*	*0*.*009*	*0*.*016*

ANOVAs were carried out for each cultivar separately. Values are means and standard deviations (SD) calculated over 12 replicates. An F test of each independent ANOVA is presented.

^a,b,c^The ear parts followed by the same letters did not differ significantly according to a Newman-Keuls post-hoc test. Y_0_ is the initial grain weight and Y_m_ the final grain weight. ns = not significant

**P<0.01

***P<0.001

In both cultivars, differences in final grain weight and in mean grain-filling rate were the main parameters distinguishing the basal, central and apical ear parts.

## Discussion

Using two bread wheat cultivars with contrasting grain-set strategies, this study characterised the growth kinetics of the grains in relation to their position within the ear and within the spikelet. The aim was to identify the main parameters driving variability in final grain weight.

### Duration of flowering within an ear

For both cultivars under our experimental conditions, the results show that about 120°Cdays (about 5.5 days) were required for all fertile florets within the ear to reach flowering. This agrees with previous studies which reported intervals of from 2 to 6 days for this [17, 24]. This finding raises the question of the differential effects of any abiotic or biotic stresses that may occur at the single grain scale around the time of anthesis. This is particularly the case for stresses whose physiological effects will depend strongly on timing. For example, Prasad and Djanaguiraman [25] demonstrated that a high temperature stress (35/25°C, day/night) applied at 6 or at 4 days before flowering, resulted in a two-fold variation in floret fertility. Clearly such a strong effect will significantly impact grain number per m^2^. However, it will also likely affect individual grain growth, and this response will depend on the developmental stage at the time of the stress event.

The 5.5 day time period over which the entire flowering of a single ear takes place, should be considered in relation to the conditions of our study. Here, only the ears originating from the main tillers were studied, also our plants were grown at a lower density than generally applies in the field. Nevertheless, Willenborg et al. [26] observed a similar range of within-ear flowering times for four different wheat cultivars under field conditions. De Vries [27] studied flowering times on up to three ears per plant, in five spring-wheat varieties under greenhouse conditions. While the main ear flowered between 2 and 4 days before the two secondary ears, the authors did not report differences in the range of flowering times within each of the different classes. Clearly, the duration of complete flowering in a single ear observed in our study, is similar to those observed in the field. It is also greater than that observed between the 2–3 larger ears, usually present in a field-grown wheat plant. This indicates that intra-ear and/or intra-spikelet variability in flowering time is a major source of total variability.

Our results show this timing variability is spatially structured and follows the axial (vertical) and lateral (horizontal) gradients in a wheat ear. This finding agrees with those of the only two earlier studies, made from a similar perspective [17, 24].

The putative effects of flowering time shift on final grain weight will now be discussed.

### Distribution of individual final grain weight according to the spikelet position along the ear

One of the aims of our study was to characterise the differences in final grain weight in relation to the two ear gradients–vertical and horizontal. As regards the two proximal grains within a spikelet (horizontal gradient), previous studies have yielded contrasting results. Some authors who worked only on grains located in the central spikelets observed heavier grains in the second position [13, 15, 19]. Meanwhile, others found the second grain was generally heavier than the first but with an interaction with spikelet position in the ear [17, 18, 21]. In particular, Li et al. [21] showed that the higher weight of the second grain is particularly marked in spikelets localised in the central position of the ear. Based on a study of three wheat cultivars, Herzog and Stamp [20] observed that a ‘standard’ wheat cultivar (as opposed to a semi-dwarf or an oligoculm cultivar) produced heavier grains in the proximal position within a spikelet. However, Feng et al. [6] found no difference in final grain weight between the two proximal grains of a spikelet in five wheat cultivars grown under field conditions. Our results are consistent with the latter result, since we found neither final grain weight nor growth kinetic differences between the two proximal grains. And this observation was independent of spikelet position along the ear (S3 Fig). Because we found no significant difference in grain weight between the two proximal grains of a spikelet, we pooled these in further analyses of grain growth in terms of spikelet position along the ear. In other words, our study was restricted to the effects of the vertical gradient.

The variability of final grain weight in relation to spikelet position along the ear has been described in different ways by different authors. Some have presented values of individual grain weight from each spikelet. In these, the average final grain weight per spikelet was found to follow a parabolic pattern along the ear with higher values in the middle spikelets [6, 21, 28]. More commonly, results have been presented based on subdivisions of the ear (as we have). This allows statistical comparison between the subdivisions. Of these studies, two reported heavier grains in spikelets in the central part of the ear but could not distinguish between apical and basal ear parts that both bore smaller grains [17, 19]. Other authors reported similar grain weights in the central and basal parts while grain weights were significantly lower only in the apical part [20]. Our results agree with Bremner [18] who recorded the average weight of the two proximal grains and with Miralles and Slafer [15] who recorded the weight only of the grain nearest the rachis. In our results, we identified statistically significant differences between the three different ear parts–the heaviest grains were in the central part, lighter grains were in the basal part and lighter grains still were in the apical part (Table 2).

### Relationship between flowering time shift of individual florets and final grain weight

Given the general similarity between the gradient of flowering and that of final grain weight, one might hypothesise a causal relationship between the two. Under this hypothesis it would seem reasonable to assume the earliest grains might benefit from lower competition for metabolites during early grain filling while only a few grains are actively developing. However, we could detect no systematic effect of flowering time shift on final grain weight. Indeed, in neither cultivar did the flowering-time difference between the first and second florets, result in a grain-weight difference, in any of the three ear parts (Fig 2; Table 2). Also, in Renan there were no differences in flowering time between the central and apical parts of the ear (Table 1). Nevertheless, significantly heavier grains were found in the central part (Table 2). We thus conclude it is unlikely the differences in final grain weight between ear parts originate from a flowering time shift. The cause of the grain weight difference between ear parts would thus seem to arise from different grain growth kinetics in each part.

### Relationship between grain growth traits and final grain weight along the ear

We studied three parameters: initial grain weight, and the duration and rate of grain filling. Hazan et al. [22] and Calderini et al. [29] hypothesised that initial grain weight at flowering (evaluated as carpel weight) is a major determinant of final grain weight. Our results do not particularly support this hypothesis, as no significant correlation (at a 5% level) between the two variables was detected for the different ear parts (r = 0.72, p = 0.11 for Apache and r = 0.52, p = 0.30 for Renan). This suggests the two other parameters may be of greater influence. Nevertheless, we cannot exclude the possibility of an indirect effect of initial grain weight on final grain weight, mediated by one of these two other parameters as discussed further below.

A number of growth kinetics studies have been conducted on average grains sampled at maturity from the central spikelets of an ear. Using different bread wheat genotypes and environments, these show that the rate of grain filling during the linear phase had a stronger effect on final grain weight than the duration of grain filling [13, 14, 16]. However, very few studies have considered the differences in grain growth kinetics based on spikelet position along the ear. For Bremner [18] as well as for Herzog and Stamp [20], the duration of grain filling was associated with the differences in final grain weight observed between the different parts of the ear. Conversely, Miralles and Slafer [15] did not find any association between the two variables. Our results are consistent with the latter authors, since we found no correlation between final grain weight and grain filling duration. It is worth noting that calculating grain filling durations from logistic curves, led to low precisions in our estimates, with standard errors of between 30 and 90°Cdays (Table 2). Our study revealed an effect of mean grain filling rate in the determinism of final grain weight differences along the ear. In both our cultivars, the mean grain filling rate in the central part of the ear was 11% higher than in the basal part, and 16% higher than in the apical part. Similar results have been found previously although no statistical comparisons were reported between the different parts of the ear [15, 20].

### Different physiological hypotheses explaining grain filling rate differences along the ear

A number of physiological hypotheses have been proposed to explain differences in grain filling rate along the ear. Xie et al. [16] pooled data from several genotypes and floret positions and found significant relationships between the initial grain weight at flowering and the rates of grain growth during both the lag phase and also during the linear phase of grain filling. This relationship could be the result of grains with higher carpel weights at flowering having higher sink strengths. We found for both cultivars that grains located in the central part of the ear had larger initial weights and also higher mean grain-filling rates. However, we also found different mean rates of grain growth in apical and basal ears parts in Apache and in central and basal parts in Renan, even though the initial grain weights were not significantly different. Even if these comparisons are qualitative in nature, they do not indicate a strong effect of initial grain weight on grain filling rate, at least when considered independently of ear part.

An alternative hypothesis to explain the different grain-filling rates along the ear may be linked to the activity of specific hormones within the grain. The importance of hormonal regulation in grain development has often been stressed, particularly in wheat (*e*.*g*. [30, 31, 32]). More importantly, several authors have shown the importance of hormonal gradients in reproductive structures and their possible link with vertical gradients of grain/seed size. Thus, working with three rice hybrids, Zhang et al. [33] showed that the low rate of filling in grains in inferior spikelets, is linked to low numbers of endosperm cells and to low contents of ABA, cytokinin and auxin during early grain filling.

A further possibility is that the structure of the ear vascular system could also contribute to differences in grain-filling rate along the ear. Whingwiri et al. [34] found that the numbers and diameters of vascular bundles decreased from the base to the tip of the ear. Thus, assimilate transport may be more limited in spikelets located near the tip of the ear. They also found that the spikelets at the base of the ear were supplied by branching bundles–branching could reduce assimilate supply. Meanwhile, spikelets in the central part of the ear are supplied by unbranched and larger bundles that may offer less resistance to assimilate transport [34].

## Conclusion

We show that individual grain weights are greatest in the central part of the ear, less in the apical part and intermediate in the basal part. These differences do not originate from a flowering time shift. However, two results are worth noticing about this study of flowering time: (i) about 5.5 days was required for all fertile florets in an ear to reach flowering; (ii) no interaction was found between the flowering time shifts within the ear, and within the spikelet. Thus, these differences of final grain weight along the ear were due mainly to differences in average grain filling rate. Unlike some other studies, we did not find clear evidence for an effect of initial grain weight on either final grain weight or on average grain filling rate.

A better understanding of the factors contributing to average grain filling rate and to final grain weight will be useful if we are to increase individual grain weights in the apical and basal parts of the ear while maintaining those in the central part. This understanding will be particularly valuable in the context of climate change, which indicate an increased occurrence of unfavourable growing conditions.

## Supporting information

S1 Fig Daily averages of environmental values recorded every 15 min from anthesis to maturity. A: Air temperature, B: Photosynthetically active radiation (PAR), C: Relative humidity.
(TIF)

S2 Fig **Frequency of floret presence according to position within the ear and spikelet for cultivars Apache (A) and Renan (B).** Ten ears were scored with 22 spikelets for Apache and with 20 spikelets for Renan.
(TIF)

S3 Fig **Effects of grain position on (a) the final grain weight and (b) the mean rate of grain filling for cultivars Apache (A) and Renan (B).** Black dot: atypical observations, black square: mean of the trait calculated on between 24 and 39 replicates.
(TIFF)

S4 Fig Effect of thermal time (°Cdays, base 0°C) on the standardised residuals from the adjusted model against observed values of grain weight. A three-parameter logistic model was fitted to each cultivar, to three ear parts and to four blocks.
(TIF)

S5 Fig The relationship between grain dry weight and thermal time after anthesis for the three different ear parts, the four blocks and for cultivars Apache and Renan. Light grey circle: apical part, dark grey circle: basal part, black circle: central part.
(TIFF)

S1 Table Summary of mean yield components between the two wheat cultivars grown in the field in 2014 and in the greenhouse in 2017. Values are means and standard deviations (SD) calculated over eight replicates for 2014 and over seven replicates for 2017. The F test of each ANOVA is presented for results obtained under field conditions.
(DOCX)

S1 Dataset Data set used to realize this study. Figs 2 and 3 and S2–S5 Figs were plotted based on this excel file. Tables 1 and 2 were created based on this excel file.
(XLSX)
