# Supplementary figures and images for: Different grain-filling rates explain grain-weight differences along the wheat ear
Source: PLoS One. 2018 Dec 31;13(12):e0209597. doi: 10.1371/journal.pone.0209597 (PMC6312219; doi:10.1371/journal.pone.0209597)

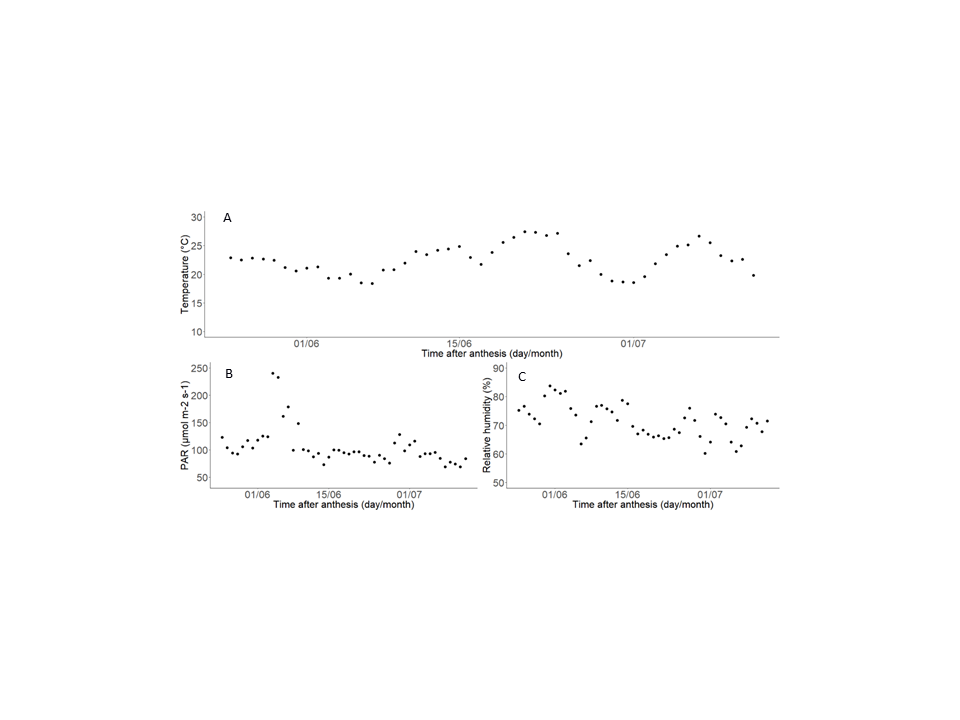

Supplement: S1 Fig — A: Air temperature, B: Photosynthetically active radiation (PAR), C: Relative humidity. (TIF) [file pone.0209597.s001.tif]

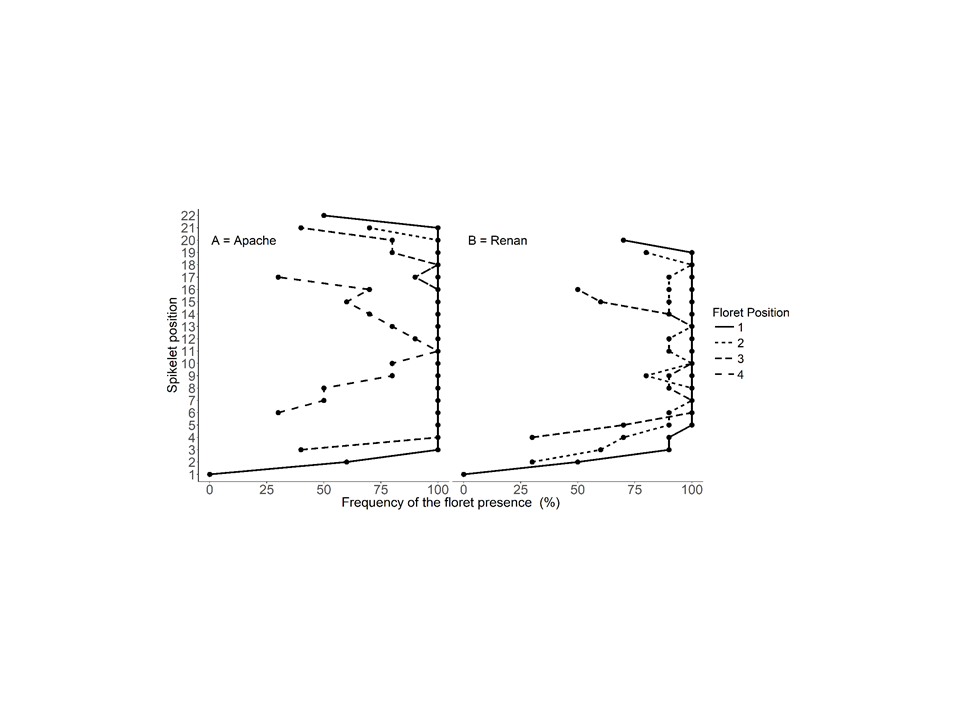

Supplement: S2 Fig — Frequency of floret presence according to position within the ear and spikelet for cultivars Apache (A) and Renan (B). Ten ears were scored with 22 spikelets for Apache and with 20 spikelets for Renan. (TIF) [file pone.0209597.s002.tif]

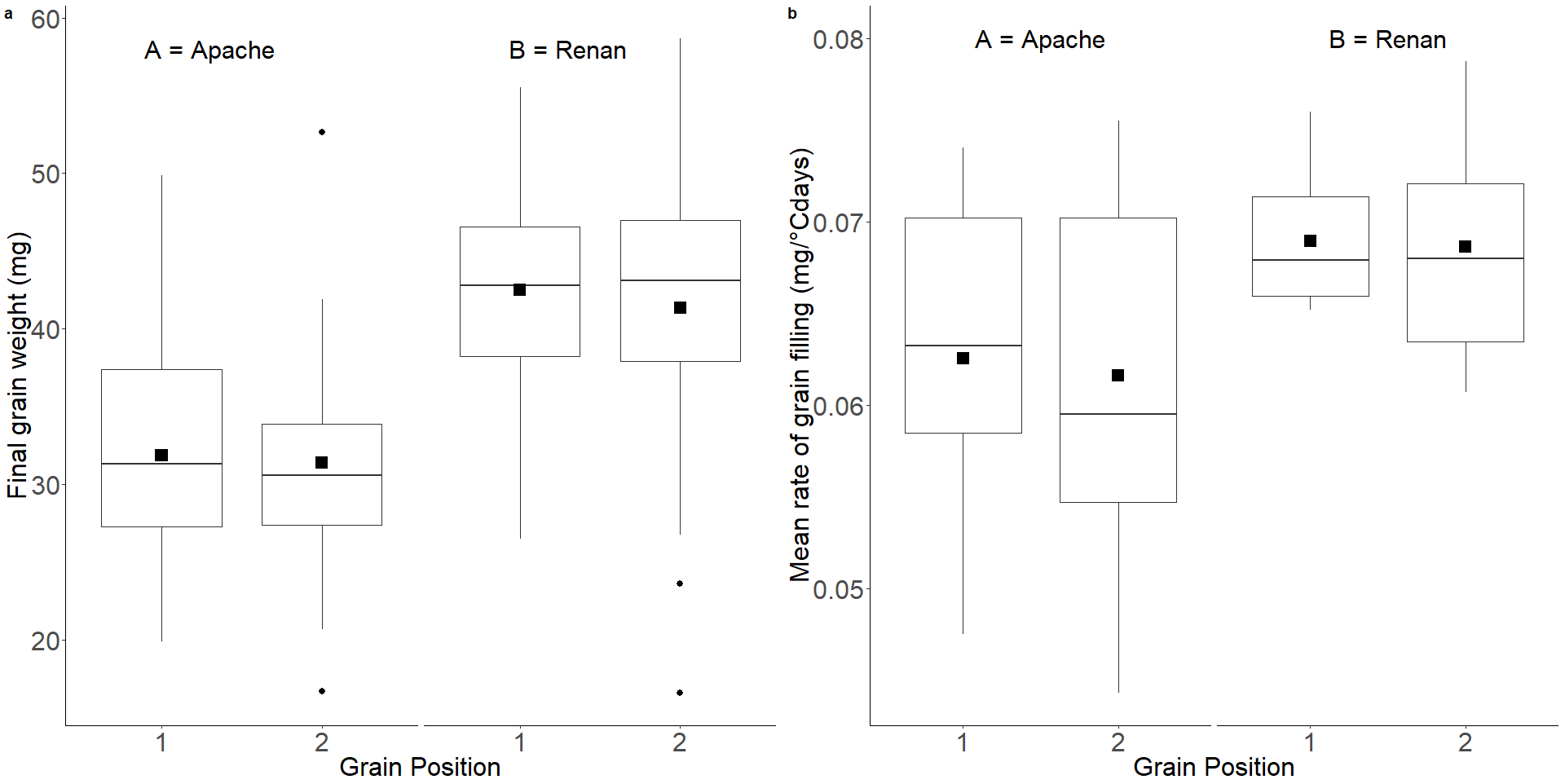

Supplement: S3 Fig — Effects of grain position on (a) the final grain weight and (b) the mean rate of grain filling for cultivars Apache (A) and Renan (B). Black dot: atypical observations, black square: mean of the trait calculated on between 24 and 39 replicates. (TIFF) [file pone.0209597.s003.tiff]

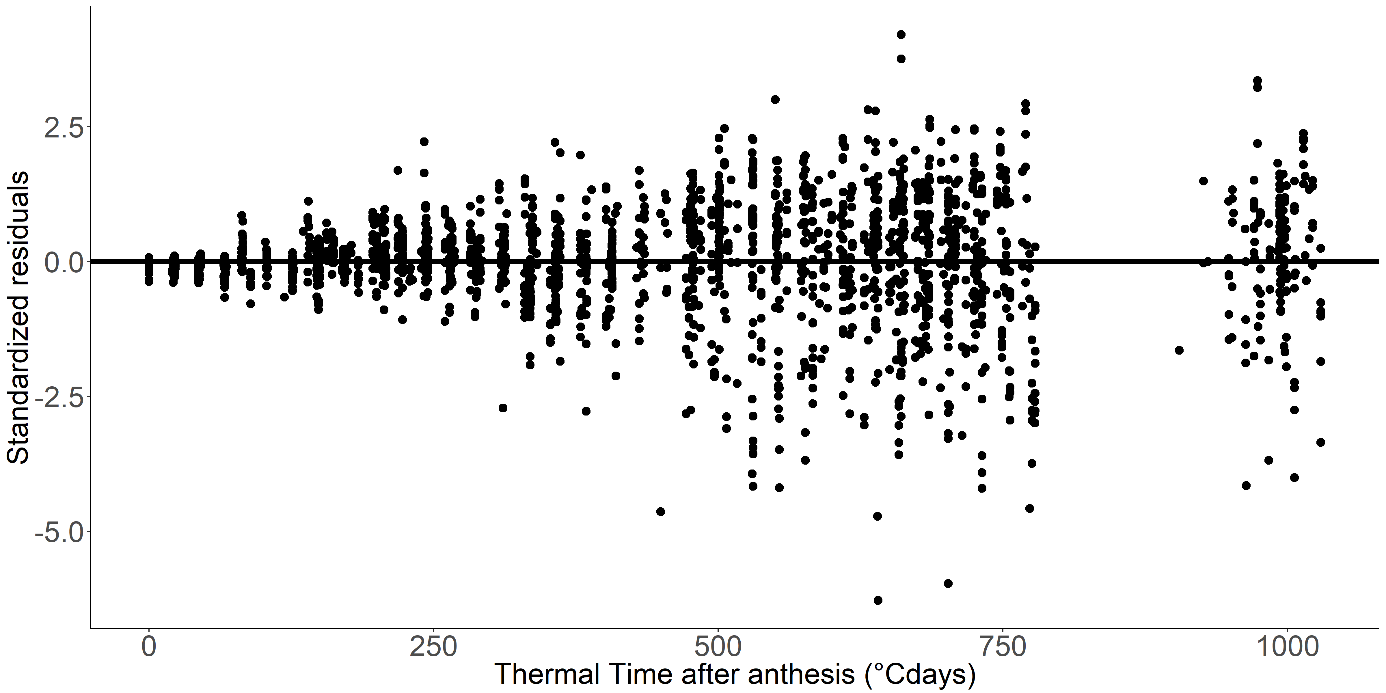

Supplement: S4 Fig — A three-parameter logistic model was fitted to each cultivar, to three ear parts and to four blocks. (TIF) [file pone.0209597.s004.tif]

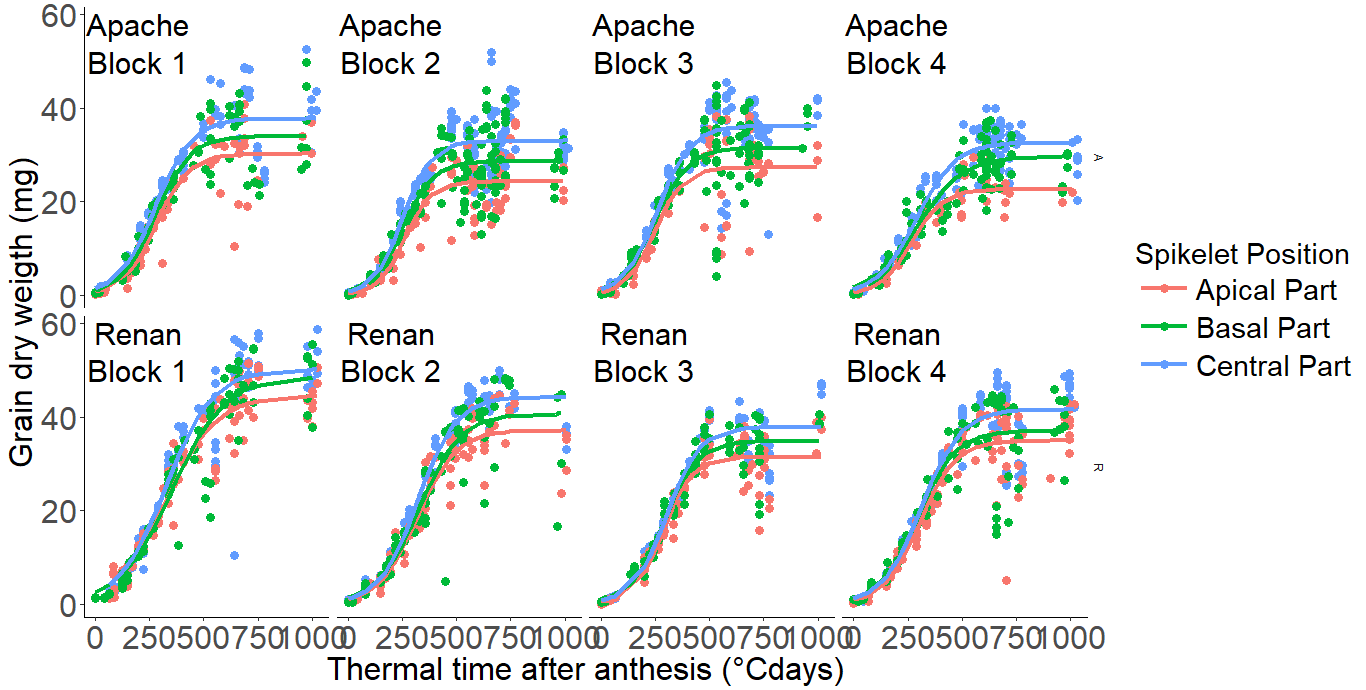

Supplement: S5 Fig — Light grey circle: apical part, dark grey circle: basal part, black circle: central part. (TIFF) [file pone.0209597.s005.tiff]
