# Supplementary material for: Different grain-filling rates explain grain-weight differences along the wheat ear
Source: PLoS One. 2018 Dec 31;13(12):e0209597. doi: 10.1371/journal.pone.0209597 (PMC6312219; doi:10.1371/journal.pone.0209597)
Supplement: S1 Table — Values are means and standard deviations (SD) calculated over eight replicates for 2014 and over seven replicates for 2017. The F test of each ANOVA is presented for results obtained under field conditions. (DOCX) [file pone.0209597.s006.docx]

| Yield components | Field 2014 | | | Greenhouse 2017 | | | |
| --- | --- | --- | --- | --- | --- | --- | --- |
|  | F(test) | Apache | Renan | F(test) | Apache | Renan |  |
| Number of ears / plant | ns | 2.1 (±0.2) | 2.0 (±0.2) | ns | 7.4 (±3.2) | 7.4 (±1.9) |  |
| Number of grains / ear | ** | 41.4 (±6.7) | 31.7 (±5.1) | ns | 34.2 (±5.3) | 32.5 (±6.2) |  |
| Number of spikelets / ear | ** | 19 (±1.1) | 16 (±0.9) | *** | 22 (±1.4) | 20 (±1.1) |  |
| Thousand grain weight (g) | *** | 41.5 (±1.2) | 47.4 (±0.6) | ns | 28.9 (±3.2) | 30.2 (±4.4) |  |
